# Synergistic Antimicrobial Effectiveness of Plant Essential Oil and Its Application in Seafood Preservation: A Review

**DOI:** 10.3390/molecules26020307

**Published:** 2021-01-09

**Authors:** Xianpei Huang, Yuli Lao, Yifeng Pan, Yiping Chen, Haiming Zhao, Liang Gong, Nanbin Xie, Ce-Hui Mo

**Affiliations:** 1 College of Life Science and Technology, Jinan University, Guangzhou 510632, China; xianpeihuang@163.com (X.H.); zhaohm99@jnu.edu.cn (H.Z.); 2 Shanwei Marine Industry Institute, Shanwei Polytechnic, Shanwei 516600, China; panyifeng1989@sina.com (Y.P.); yipingchen2020@163.com (Y.C.); nanbinxie@126.com (N.X.); 3 College of Life Science, University of Chinese Academy of Sciences, Beijing 100049, China; yuli.lao@foxmail.com; 4 Key Laboratory of South China Agricultural Plant Molecular Analysis and Genetic Improvement, South China Botanical Garden, Chinese Academy of Sciences, Guangzhou 510650, China; lianggong@scbg.ac.cn

**Keywords:** plant extract, sea product, antimicrobial agent, foodborne pathogen, synergistic effect

## Abstract

The synergistic potential of plant essential oils (EOs) with other conventional and non-conventional antimicrobial agents is a promising strategy for increasing antimicrobial efficacy and controlling foodborne pathogens. Spoilage microorganisms are one of main concerns of seafood products, while the prevention of seafood spoilage principally requires exclusion or inactivation of microbial activity. This review provides a comprehensive overview of recent studies on the synergistic antimicrobial effect of EOs combined with other available chemicals (such as antibiotics, organic acids, and plant extracts) or physical methods (such as high hydrostatic pressure, irradiation, and vacuum-packaging) utilized to reduce the growth of foodborne pathogens and/or to extend the shelf-life of seafood products. This review highlights the synergistic ability of EOs when used as a seafood preservative, discovering the possible routes of the combined techniques for the development of a novel seafood preservation strategy.

## 1. Introduction

**Figure 1:**
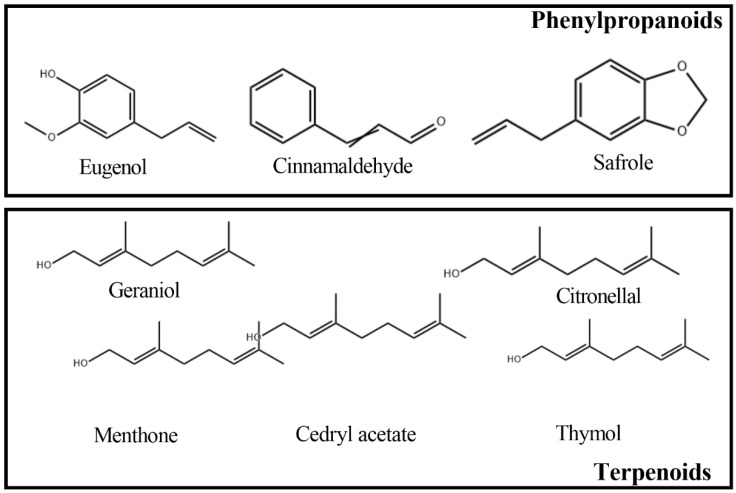
Chemical structural formulas of some essential oils.

The commonly used antimicrobial agents include two types: Natural and synthetic compounds. With the increasing concerns for food safety and the understanding of the harm of synthetic preservatives to human health on multiple fronts, research of natural preservatives has recently been significantly developed in the field of seafood preservation [1,2], as it is known that seafood is a rich nutrient source for microbial development and very prone to the loss of quality caused by spoilage microorganisms [3]. Essential oils (EOs) are mixtures of volatile and hydrophobic substances from plants, which can be considered ubiquitous in the plant kingdom. While some of them with promising antimicrobial and antioxidative bioactivities extracted from various genera distributed in approximately 60 families, which are known to produce EOs with high application value in the pharmaceutical and cosmetic industries, mainly including *Alliaceae*, *Apiaceae*, *Asteraceae (Compositae)*, *Lamiaceae (Labiatae)*, *Myrtaceae*, *Poaceae*, *Cupressaceae*, *Lauraceae*, *Pinaceae*, *Zingiberaceae*, and *Rutaceae* [4]. EOs are organic volatile compounds, generally the molecular weight is less than 300 Da, and they are composed of two distinct chemical classes including terpenoids and phenylpropanoids, as some examples are shown in Figure 1, and after extensive research, it is known that many compositions of EOs have excellent antimicrobial and antioxidative properties [5]. Among these active compounds, small molecule phenols or aldehydes contributed greatly to the antimicrobial effect, such as carvacrol, cinnamaldehyde, and cuminaldehyde [6]. Additionally, terpene alcohols, ethers, and ketones also have certain antimicrobial activities, such as carvone, geraniol, and γ-tepinyl acetate [7]. Current research has demonstrated that one of the most popular proposed mechanisms is that the action of EOs leads to the breakdown of the microbial membrane, resulting in the leakage of cellular components, loss of ions, and prevention of many cellular life activities, including energy production and cell membrane transport [8].

When two or more compounds are used at the same time, the mixtures with the antagonist result in the chemical substance being antagonized, and if the effectiveness is smaller than that of one of them, it is regarded as antagonism. When the comprehensive effect of the mixture is greater than the sum of the individual actions, it is considered to be a synergistic effect, while an additive effect is defined as the effects of the combinations are equal to the sum of the effect of each chemical substance. Actually, it is possible to combine several antimicrobial compounds to create a synergistic or additive effect. The main objective of the combined techniques in the food industry is to reduce the growth and activity of spoilage microorganisms more efficiently in food products to increase their shelf-life and reduce the losses from decay, with the final aim to protect food safety and quality [9]. The spoilage of seafood could be caused by microbial infection, autolysis, or chemical oxidation, while microbial infection constitutes more spoilage than the others. Microorganisms involved in seafood spoilage mainly include the spoilage bacteria *Alteromonas nigrifaciens*, *Shewanella putrefaciens*, *Brochothrix thermosphacta, Aeromonas hydrophila, Photobacterium phosphorous*, and *Pseudomonas spp* and foodborne pathogens, such as *Listeria monocytogenes*, *Vibrio* spp., and *Salmonella* spp. [10]. In addition, in seafood products bacterial growth and metabolic activity cause the formation of ammonia, biogenic amines, organic acids, sulfur compounds, and ketones, which are responsible for unpleasant and unacceptable off-flavors [11]. On the other hand, contaminated seafood can cause harm to human health, as seafood is an important reason for the outbreak of foodborne disease. In the EU, seafood accounted for more than 10% of foodborne outbreaks in 2015 [12], and the CDC estimates that approximately 48 million people are affected and 3000 deaths occur each year due to foodborne illness in the United States [13]. Second, pathogens such as *Vibrio* and norovirus in shellfish are the major causes of seafood-mediated diseases, such as diarrhea and vomiting [14]. Enhanced disinfection technology is therefore needed to improve the safety of seafood for human consumption. In this regard, recently, physical, chemical, and biological methods have been developed and used to reduce or inactivate the growth and survival of seafood spoilage bacteria and foodborne pathogens to extend the shelf-life of seafood products and protect human health. EOs and their bioactive constituents have attracted great attention because of their potential functions and properties for use in the seafood industry, particularly recently, as we have an increased threat of antimicrobial resistance to common preservatives. Fortunately, the synergistic or additive antimicrobial effects of EOs or their components combined with chemical or physical methods have been intensively investigated in the past five years [15,16].

This review paper contains five sections. The Section 1 is the introduction. The Section 2 summarizes the synergistic or additive effects of the combination of EOs with other chemicals, such as antibiotics, organic acids, and plant extracts. The Section 3 reviews the active edible coatings incorporating EOs as synergistic factors. In the Section 4, we discuss the preservation techniques using physical devices, such as high hydrostatic pressure, irradiation, and vacuum-packaging, and their applications in the seafood industry. The Section 5 discusses the further applications of the combined techniques using EOs as one of the main methods in seafood preservation.

## 2. Synergistic Effect of Essential Oils with Other Compounds on Seafood Preservation

Compared to the use of only an EO, the combination of an EO with other bioactive compounds, including antibiotics, organic acids, and other EOs, may have a stronger antimicrobial activity compared to those of any single compound, which can be attributed to the mixture increasing the diversity of components and resulting in multiple sites of action [17,18]. As mentioned previously, seafood is very perishable, and food-borne pathogens in seafood not only lead to spoilage, but also cause serious illnesses that are harmful to human health. *Salmonella* bacteria, the most frequently reported cause of foodborne illness, has been linked to contaminated shrimp [19] and other seafood [20]. Nevertheless, EOs combined with other plant extracts showed synergistic antimicrobial effectiveness against food-borne pathogens. For example, Porter et al. (2019) reported MICs (minimum inhibitory concentrations) of 0.5, 0.02, and 0.02% for white mustard essential oil (WMEO), carvacrol, and thymol, respectively, against *S.* Typhimurium, while WMEO combined with thymol or carvacrol indicated that the concentration of the individual essential oil was reduced in the serovars test for controlling *Salmonella* [21]. It was also found that the combined effect of *Trachyspermum ammi* EO and propolis ethanolic extract increases the antimicrobial efficacy against some food-borne pathogenic bacteria, including *Escherichia coli*, *S.* Typhimurium, *L. monocytogenes*, *Bacillus cereus*, and *Staphylococcus aureus* [22]. Additionally, mustard EO also exhibited synergistic effects against foodborne pathogenic bacteria when combined with either *Mexican oregano* or thyme EO [23]. In addition, plant extracts have also been used for food preservation and to extend the shelf-life of seafood products. For example, the combination of turmeric powder (1.0%), galangal powder (1.25%), and lemongrass essential oil (0.5%) created a synergistic effect that could effectively maintain the physico-chemical, microbial, and sensory characteristics of white hard clam muscle for 12 days of storage [24].

The application of antibiotics is a common practice for control food-decaying microorganisms and human pathogens. However, the long-term and extensive use of antibiotics leads to a series of problems, such as residue and environmental pollution, the resistance of bacteria, and human health problems. Moreover, it requires increasing the dosage to treat the antibiotic-resistant disease, which in turn creates a vicious circle. EOs are also alternative compounds to control multidrug-resistant pathogens. Furthermore, synergistic antimicrobial activity against foodborne pathogens was revealed when antibiotics were used in combination with EOs. de Jesus et al. (2020) reported that EOs from Cerrado plants proved to be active against resistant bacteria including *S. Typhi* and oxacillin-resistant *Staphylococcus* strains [25]. Clove and thyme essential oils exhibit antimicrobial activities that have been mainly attributed to their ability to inhibit bacterial biofilm formation, and hence, they have been proposed as a natural alternative to be used in combination with antibiotics against multidrug-resistant bacteria [26]. In addition, Fennel essential oil mainly composed of *trans-anethole* (80%) was found to significantly enhance the inhibition zone against resistant phenotypes of *S. aureus in the combined treatment with* cefoxitin, mupirocin, cotrimoxazole, and ciprofloxacin [27]. Pereira et al. (2017) reported that Eugenia uniflora essential oil (EuEO) presented a synergistic efficiency associated with amikacin against *S. aureus* and *E. coli, while an exception was observed when* EuEO was combined with *gentamicin against E. coli, in which an antagonistic effect was observed* [28].

Organic acids, such as citric acid (CA), lactic acid (LA), and capric acid (CP), are ‘generally recognized as safe’ (GRAS), as they occur naturally in foods and are particularly suitable for the tread of green consumerism, which encourages food processors to find alternatives to synthetic chemicals for food preservation. Organic acids and their salts were found to have preservative activity in seafood, and the activity can be increased when used in appropriate combination with EOs. Recently, it has been confirmed that mixtures of tartaric acid and garlic oil (or lactic acid and cinnamon oil) are effective in extending the shelf-life of raw shrimp, as indicated by both their microbiological and physicochemical properties [29]. Dogruyol et al. (2020) reported that the thermal sensitivity of *L. monocytogenes* was increased in sous-vide salmon by the combined treatment of oregano essential oil and citric acid [30].

Spoilage is a major problem in the transportation and selling of fish due to this issue of their limited shelf-life. Thus, methods for fish preservation to prevent quality deterioration and microbial growth are needed. Recently, the comprehensive use of food-grade compounds and EOs to control fish spoilage has achieved a better control effect. As also listed in Table 1, recent studies have described the synergistic antibacterial activity of EOs combined with other food-grade chemicals utilized for the control of seafood spoilage. In addition, it would be interesting to consider the effect of EOs treatment on the gustatory properties of seafood, and this may be widely accepted because people cook seafood often accompanied by the use of plant materials (such as lemon oils). However, the concentrations of EOs should not exceed the acceptability of human taste.

## 3. Synergistic Effect of EOs with Edible Coatings and Films for Seafood Preservation

EOs are an original pharmaceutical plant resource with promising antibacterial and antioxidant properties. However, their hydrophobic, volatile, strong sensory, UV-sensitive, etc., natural attributes are major impediments in the use of EOs for food preservation. Methods developed to overcome these shortcomings mainly include nanoemulsions or biofilms. Nanoemulsions with a droplet size between 5 and 200 nm are extensively used for the delivery of hydrophobic EOs, which commonly consist of water, oil, and an emulsifier [36]. Meanwhile, the application of EO-based nanoemulsions either as a washing disinfectant or incorporated into edible coatings has been shown to increase the efficacy when combined with other handling techniques [37,38]. On the other hand, active packaging films or antibacterial packaging films have greatly improved the stability of EOs and broadened the path for their application in food preservation. For example, EOs can be used to improve the properties of potato starch-based films and extend the shelf-life of shrimp [39]. Carvacrol incorporated in flaxseed gum films can significantly reduce the TVB-N content as well as the degree of microbial deterioration and improve the quality of Chinese sea bass during cold storage [40].

Edible coatings and films used as a new technology for food packaging are applied directly by forming an edible thin layer on the surface of the food product, and this system has some excellent properties, such as edibility, biocompatibility, and barrier properties. To make better use of edible coatings and films for fishery products, the jointed use of new natural inhibitors and coating carriers for their synergistic or additive effects has been substantially studied (Table 2). Particularly, edible coatings and films consist of biopolymers, such as polysaccharides, protein, and lipids, which is quite promising for their application in seafood preservation, and they are also suitable packaging materials to integrate EOs for use as biodegradable materials. For example, bombacaceae gum incorporated with cinnamon leaf essential oil exhibited antimicrobial activity and antioxidation of lipids in fresh salmon fillets during refrigerated storage [41]. Pectin coatings enriched with clove essential oil can extend the shelf-life of bream fillets by at least 15 days [42].

Recently, biopolymer-based biodegradable functional films have been found to have great potential for use in food packaging applications. Chitosan, known as a polysaccharide, is derived from chitin, which is rich in the exoskeleton of crustaceans. Chitosan-based films have excellent characteristics, such as antimicrobial activity, barrier properties, and antioxidant activity. Recent advances in the applications of EO-chitosan-based films have been largely reported in the field of seafood preservation [43]. For example, chitosan, in combination with oregano, can significantly reduce the counts of mesophilic bacteria, *Pseudomonas*, *Shewanella*, and yeast in eel fillets stored in vacuum packaging at 4 °C for 18 days [44]. It is reported that a chitosan-whey protein composite edible coating containing tarragon *Artemisia dracunculus* essential oil can improve the deterioration of *Scomberoides commersonnianus* fillets in refrigerated conditions (4 ± 1 °C) and control the growth of mesophilic bacteria and psychrotrophic bacteria [45]. In addition, the combination of chitosan and clove EOs can inhibit the chemical and microbial deterioration of frozen tambaqui fillets for 120 days [46]. It has also been reported that chitosan, in combination with thyme oil, has the highest bioactivity for inhibiting the growth of mesophilic bacteria and extending the shelf-life of smoked eel fillets over 49 days under VP conditions and refrigeration (4 °C) [47].

In addition to chitosan, other polysaccharide, protein, and lipid-based coatings have also shown promising results. Marjoram essential oil (MEO) was incorporated into alginate/clay nanocomposite films, which were applied to control *L. monocytogenes* on trout fillets in refrigerated storage (4 °C) for 15 days, and the results showed that the nanocomposite film containing MEO more efficiently inhibited the growth of *L. monocytogenes* during the 15 day storage period, with final counts reaching 6.23 log CFU/g, while the counts in control samples were significantly higher, reaching 7.38 log CFU/g [48]. The application of pectin coatings with clove essential oil significantly improved the weight loss, water evaporation, and textural and color deterioration of the bream samples compared to the untreated sample, and the composite film showed synergistic action on bacterial growth, especially for gram-negative bacteria during refrigeration over a period of 15 days [42].

Most biopolymers used in edible coatings and films are edible biopolymers or food-grade materials. It can be considered to be eco-friendly and a food safety measure to preserve fishery products, which has the great benefit of providing “green labeling” for the final fishery product to meet the consumer requirements of a system free of chemical-synthesized preservatives.

## 4. Synergistic Effect of Essential Oils with Physical Methods for Seafood Preservation

Consumers have raised the requirements for seafood not only with regards to the quality and nutrients, but also with regards to food security, which urges the development of green processing techniques for seafood. It is, therefore, necessary to control seafood spoilage using innoxious and no residue methods. Physical disinfection technologies have been developed during the 20th century, and by their mechanism of action, they can be divided into direct or indirect effects on the inactivation of pathogenic microorganisms. Treatments such as heating, ultraviolet (UV) light, or some thermal or nonthermal processes can directly kill or inhibit the growth of spoilage microorganisms, and these methods also show synergistic antimicrobial effectiveness against food pathogens in the combined treatments with EOs. For example, citrus lemon (CLEO) and C. reticulata oils (CREO) combined with heat treatment affected almost the entire cell population (up to 99%) of *Levilactobacillus brevis* and *Leuconostoc mesenteroides*, while with exposure to CLEO and CREO alone, the cell populations with physiological damage were in a range of 19.6–66.8% and 23.8–75.9%, respectively [54]. Synergistic antimicrobial effects generated by UV light and EOs were also reported, and it has been demonstrated that UV-C light enhanced the antibacterial activity of clove oil against *S.* Typhimurium by inactivating the biofilms on stainless steel [55]. High hydrostatic processing (HPP) is one of the extensively explored nonthermal food processing technologies, which has great potential to preserve the freshness of food without affecting the food quality characteristics, such as the color, natural flavor, and nutrients, in addition to directly killing fish-spoiling bacteria, such as *L. monocytogenes*, *E. coli*, and *V. parahaemolyticus*. Furthermore, the synergistic effect of HPP with EOs has also been demonstrated. Kung et al. (2020) reported that HHP in combination with 0.2% lemon essential oil was more effective under the same pressure to inactivate *Morganella morganii*, a histamine-forming bacteria [56]. The combined treatment of HPP and carvacrol has also shown synergistic inactivation effects on *Salmonella* and *L. monocytogenes*, while only HPP-induced bacterial injury can be recovered [57]. In another interesting study, chitosan films with 20 g kg^−1^ clove essential oil were combined with HPP processing as preservative treatments for trout fillets and showed a significant additive effect on the inhibition of growth of aerobic mesophilic and coliform counts when stored at 4 °C for 22 days [49].

**Figure 2:**
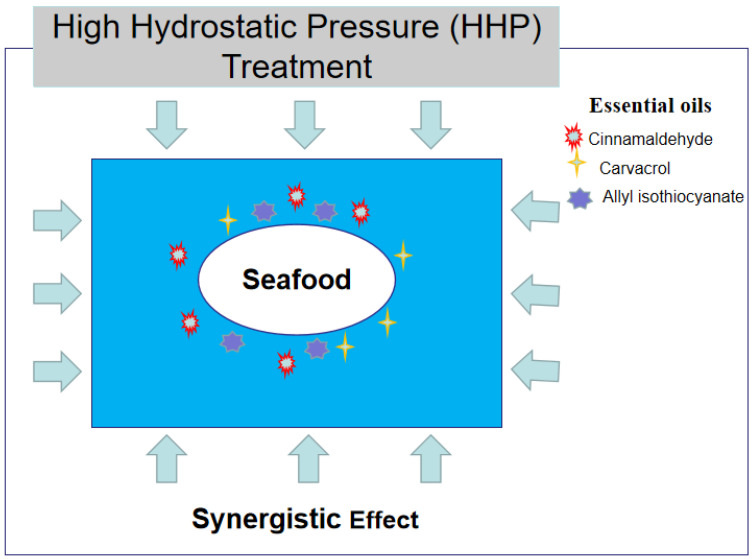
Combined treatment of essential oils and high hydrostatic pressure technique have synergistic inactivation effects on pathogenic and spoilage organisms in seafood.

Another technique has the indirect function of a physical machine to regulate the environment inside the pack in order to maintain seafood quality, such as modified atmosphere packaging (MAP) and vacuum packaging (VP). Both can be considered as packaging techniques, but can be accomplished by coupling with a physical machine. MAP and VP create different gaseous atmospheres surrounding a food product inside a pack. MAP provides alterations of atmospheric gas concentrations in the pack involving the three principal gases, including carbon dioxide, nitrogen, and oxygen, while in VP, the air is completely removed. Both are usually used for seafood preservation in the presence of EOs. Oregano EOs in the vapor phase can enhance the effectiveness under MAP conditions (60:40 CO_2_/N_2_) to extend the shelf-life of fish fillets to at least 28 days (4 °C), while the quality declined on day 7 in untreated fillets [58]. In addition, corn zein-based edible coatings incorporating nisin and lemongrass essential oil (8%) have been suggested to control the spoilage bacteria *L. monocytogenes* on cold-smoked sunshine bass and extend the shelf-life to 14 days and 42 days with polyvinyl chlorine (PVC) and VP treatments, respectively, at 4 °C [59]. The addition, R(+) limonene could extend the shelf-life of gilt-head sea bream fillets packaged in vacuum conditions until 15 days of storage without any significant loss of texture, odor, and color [50]. It has been reported that chitosan in combination with thyme oil has the highest bioactivity to inhibit the growth of mesophilic bacteria and extend the shelf-life of smoked eel fillets over 49 days under VP conditions and refrigeration (4 °C) [47]. Chitosan films mixed with pink pepper residue extract and combined with modified atmosphere packaging (100% CO_2_) were highly effective in maintaining the quality properties of skinless salmon fillets during refrigerated storage for 28 days [60]. As summarized in Table 3 and Figure 2, MAP, VP, or HHP combined with EOs could be regarded as effective and eco-friendly techniques to preserve the quality and safety of fishery products.

## 5. Perspectives

**Figure 3:**
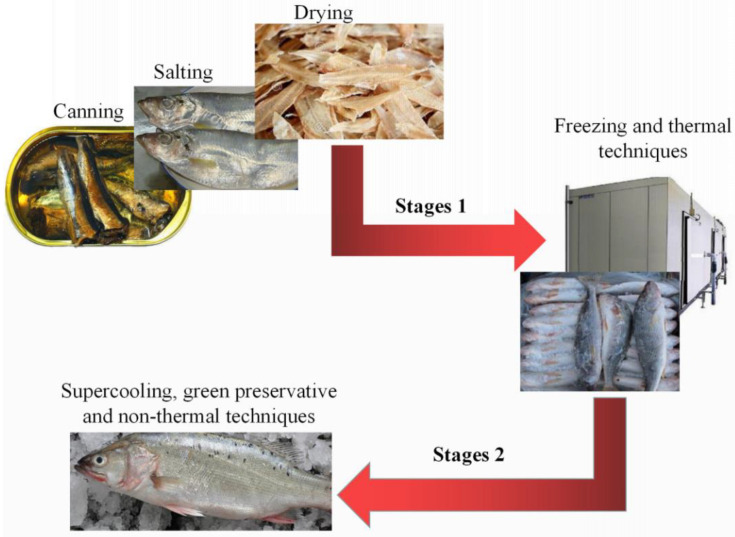
The seafood preservation technology has been developed from the first stage to the second stage, and emerging novel preservation techniques are more effective to extend the shelf-life of fresh seafood products and minimize their nutritional losses.

The traditional preservation methods, such as salting, drying, smoking, fermentation, and canning, have been replaced by the modern methods, such as supercooling, freezing, chemical preservation using food-grade compounds, edible films and coatings, HHP, and other nonthermal processes, which are more efficient and more environmentally friendly compared to the historical handling techniques (Figure 3). Moreover, EOs mainly consist of volatile components obtained from special plant materials, which can be added to various preservative approaches to improve the antimicrobial and antioxidant properties. The comprehensive use of these modern preservation techniques can significantly extend the shelf-life of fish and fulfil the consumer’s requirements regarding its texture, appearance, and taste.

Fish and other seafood are highly perishable due to the combined action of spoilage caused by bacterial and chemical degradation, as well as enzymatic and mechanical damage. Using edible films/coatings containing EOs combined with cooling and freezing, or further linking them with vacuum packaging seems to be the most effective synergistic method to maintain the quality of seafood and prevent the microbial activity. Future developments in seafood preservation techniques should consider the fish spoilage mechanisms, synergistic antimicrobial mechanisms, optimization of the environmental conditions, minimization of temperature fluctuations, the initial microbial type and load, and their interaction effects in order to optimize the shelf-life of fishery products.

## Figures and Tables

**Table 1:** Synergistic or additive effect of essential oils combined with other chemical substances on quality retention of seafood products.

Plant Sources	Combined Chemical Compounds	Tested Seafood	Effectiveness	Reference
*Origanum* *vulgare*	Citric acid	Salmon	The lowest D- and z-value in the combined group	[30]
*Monarda punctate*	Sodium alginate	Carp fillet	Reducing the microbial contamination about 1.5 log CFU/g	[31]
*Cymbopogon citratus*	Tumeric and galangal powder	White hard clam	Improving overall acceptability of *Meretrix lyrata* during 12 days of chilled storage	[24]
*Curcuma longa*	Sodium chloride	Tengra fish	Showing the acceptable fresh condition at the end of 12 months	[32]
*Cinnamomum cassia Presl* and *Allium sativum*	Tartaric acidLactic acid	Shrimp	Improving microbiological and physicochemical qualities of whole shrimps stored at 4 °C	[29]
*Zataria* *multiflora*	Liposome	Rainbow trout fillet	Showing the most suitable outcomes in term of antioxidant and microbial properties as well as sensory evaluation	[33]
*Citrus aurantium*	Beta-hydroxytoluene	Carp	Reducing chemical deterioration and lipid oxidation in the fillets	[34]
*Carvacrol*	Caprylic acid	Shrimp	Enhancing the efficacy of the chitosan-carvacrol coating on the quality of shrimp during 10 days of iced storage	[35]

**Table 2:** Synergistic or additive effect of essential oils combined with active packing films on quality retention of seafood products.

Plant Sources	Combined with Edible Coatings	Tested Sea Food	Effectiveness	Reference
*Origanum* *majorana*	Alginate/clay	Trout slices	Significantly delayed the growth of *Listeria monocytogenes* during the 15-day storage	[48]
*Artemisia dracunculus*	Chitosan/whey protein edible coating	Scomberoides commersonnianus fillet	Promising antimicrobial inhibition activity against TMC and PTC bacteria during storage at refrigerator for 16 days	[45]
*Syzygium* *aromaticum*	Chitosan films/high-pressure (HPP)	Trout fillets	Significant additive effect on the growth of aerobic mesophilic and coliform counts when stored at 4 °C for 22 days	[49]
*Syzygium* *aromaticum*	Pectin coating	Bream fillets	Prolonging the shelf life to 15 days during refrigeration	[42]
*Cinnamomum* *cassia Presl*	Chitosan coating/vacuum packaging(VP)	Eel fillets	>18 days for the E-CH and E-CH-OR fillets stored in VP at 4 °C	[45]
*Citrus*	R(+) limonene/vacuum packaging	Bream	Maintain shelf life bream fillets until 15 days of storage at 2 °C without any significant loss of overall acceptability	[50]
*Thymus mongolicus*	Chitosan/vacuum packaging	Smoked eel fillets	The shelf-life over 49 days in chitosan-thyme treated samples	[47]
*Syzygium aromaticum* and *Cinnamomum cassia Presl*	Starch edible film	Shrimp	Extending the shelf life to 14 and 12 days for storage at 10 and 4 °C, respectively	[51]
*Artemisia dracunculus*	Chitosan-whey protein coatings	Scomberoides commersonnianus fillets	Extending the shelf life of the fillet to 12 days at refrigerated condition	[45]
*Syzygium* *aromaticum*	Chitosan coating	Colossoma macropomum fillets	Significantly delayed lipid oxidation and inhibited the growth of culturable psychrotrophic bacteria	[46]
*Ferulago* *angulata*	Chitosan coating	Rainbow trout fillets	The shelf life of rainbow trout fillets was extended to 16 days at 4 °C	[52]
*Zingiber* *officinale Rosc* *. and Cinnamomum cassia Presl*	Alginate	Oreochromis niloticus	Showing higher sensory acceptability regarding to odor, color, and weight in treated samples	[53]

**Table 3:** Synergistic or additive effect of essential oils combined with physical methods on quality retention of seafood products.

Plant Sources	Combined Physical Method	Tested Fish	Effectiveness	Reference
*Citrus*	High hydrostatic pressure	Tuna meat slurry	Lower the D values in the combined treatments for inactivation of *Morganella morganii*	[51]
*Cinnamomum* *cassia Presl*	Pilot-plant packaging (high vacuum)	Bream fresh fillets	The lowest growth of Enterobacteria/lactic acid bacteria after 28 days at 4 °C	[58]
*Citrus* or *Lavandula angustifolia Mill*	Vacuum packing	Anchovy	Reducing the formation of biogenic amine and lowering the risk of histamine in fish	[61]
*Citrus*	Vacuum packing	Bream fillets	Resulting in a shelf-life extension of 6–9 days for treated fish	[50]
*Thymus mongolicus Ronn*	Vacuum packing	Eel fillets	Longer shelf-life to 42 days compared to the control samples of 35 days	[47]
*Syzygium* *aromaticum*	Vacuum packing	Catfish fillets	More effective in protecting the textural quality and retarding lipid oxidation	[62]
*Cinnamomum* *cassia Presl*	Vacuum packing	Carp	Decreasing the relative abundance of TVB-N, biogenic amines, and *Macrococcus* spp. in treated samples	[63]

## Data Availability

No new data were created or analyzed in this study. Data sharing is not applicable to this article.
